# Supplementary material for: The Burden of Urinary Tract Infections on Quality of Life and Healthcare in Patients with Interstitial Cystitis
Source: Healthcare (Basel). 2023 Oct 18;11(20):2761. doi: 10.3390/healthcare11202761 (PMC10606447; doi:10.3390/healthcare11202761)
Supplement: Supplementary file 1 [file healthcare-11-02761-s001.zip › healthcare-2578647-supplementary.pdf]

Diagnose en symptomen

1. **Heeft u Interstitiële Cystitis ofwel het Blaaspijn Syndroom? (IC/BPS)**
   - ☐ Ja
   - ☐ Nee, mijn partner of iemand die dicht bij mij staat heeft IC/BPS
   - ☐ Anders, namelijk……………………………………………………………………

   Toelichting: indien u deze vragenlijst invult als naaste, kunt u bij alle volgende vragen ‘u’ lezen als ‘uw naaste’.

2. **Welke klachten heeft u van IC/BPS? Vink deze aan, en geef vervolgens aan welke klacht voor u als eerst aangepast zou moeten worden. U kunt uw klachten op volgorde zetten (1, 2, 3, 4).**

	volgorde
☐ Pijn in de blaas en/of bekken	☐
☐ Frequent plassen	☐
☐ Aandrang van plassen	☐
☐ Slecht slapen door vaak plassen ’s nachts of door pijn	☐

3. **Hoe lang geleden bent u (ongeveer) gediagnosticeerd met IC/BPS?**
   - ☐ 0 - 2 jaar geleden
   - ☐ 3 - 5 jaar geleden
   - ☐ 6 - 10 jaar geleden
   - ☐ 11 - 20 jaar geleden
   - ☐ Langer dan 20 jaar geleden
   - ☐ Weet ik niet
   - ☐ Niet van toepassing/ geen diagnose gesteld

4. **Hoe lang had u al IC/BPS klachten vóór de diagnose gesteld werd?**
   - ☐ 0 - 6 maanden
   - ☐ 7 - 12 maanden
   - ☐ 1 - 2 jaar geleden
   - ☐ 3 - 5 jaar geleden
   - ☐ 6 - 10 jaar geleden
   - ☐ 11 - 20 jaar geleden
   - ☐ Langer dan 20 jaar geleden
   - ☐ Weet ik niet
   - ☐ Niet van toepassing/ geen diagnose gesteld

5. **Is de diagnose IC/BPS door een uroloog gesteld met een cystoscopie onderzoek?**
   - ☐ Ja
   - ☐ Nee — doorgaan naar vraag 7

6. **Beantwoord deze vraag alleen Indien u bij de vorige vraag ‘ja’ heeft geantwoord.**
   Wat waren de bevindingen van het blaasslijmvlies tijdens het cystoscopie onderzoek?
   - ☐ Er zijn rode zweren gevonden (de laesies van Hunner)
   - ☐ Er is een ontstoken blaaswand gevonden, maar geen rode zweren (laesies van Hunner)
   - ☐ Er zijn geen afwijkingen gevonden van het blaasslijmvlies
   - ☐ Weet ik niet

7. Mijn huisarts heeft nu of in het verleden mijn klachten van IC/BPS verward met een bacteriële blaasontsteking.

☐ Ja
☐ Nee doorgaan naar vraag 9

8. Beantwoord deze vraag alleen Indien u bij de vorige vraag 'ja' heeft geantwoord.
In welke mate heeft deze verwarring door de huisarts een vertraging opgeleverd in het krijgen van de juiste diagnose van IC/BPS.

☐ Ongeveer 6 maanden
☐ Ongeveer 1 jaar
☐ Ongeveer 2 jaar
☐ Ongeveer 3 jaar
☐ Ongeveer …………………….. jaar (zelf invullen, geheel getal)

9. Heeft u naast uw IC/BPS ook regelmatig last van een bacteriële blaasontsteking?

☐ Ja
☐ Nee doorgaan naar vraag 13
☐ In het verleden was hier sprake van
☐ Weet ik niet

10. Hoe vaak heeft u de afgelopen 2 jaar een antibiotica kuur gekregen voor een bacteriële blaasontsteking?

☐ Geen
☐ 1 tot 3 keer
☐ 4 tot 5 keer
☐ 6 tot 10 keer
☐ Meer dan 10 keer

11. Heeft u de afgelopen jaren een periode antibiotica als onderhoudsdosering gebruikt ter voorkoming van een bacteriële blaasontsteking?

☐ Ja
☐ Nee

12. Is er bij u in een urinekweek ooit een bacterie gevonden die resistent was voor een antibiotica?

☐ Ja
☐ Nee
☐ Weet ik niet

13. Kunt u hieronder aangeven op de lijn van 0 - 10 in hoeverre bacteriële blaasontstekingen uw klachten van IC/BPS negatief beïnvloeden (0 = geen invloed, 10 is zeer veel invloed).

Cijfer ……..

0 ———————————————— **10**

14. Geef aan in hoeverre u het eens bent met de volgende stelling:
'Ik ben bang voor het krijgen van een nieuwe bacteriële blaasontsteking'.

- ☐ Mee eens
- ☐ Matig mee eens
- ☐ Onduidelijk
- ☐ Matig mee oneens
- ☐ Mee oneens

15. Geef aan in hoeverre u het eens bent met de volgende stelling:
'Na het doormaken van een bacteriële blaasontsteking krijg ik in de periode erna veel meer klachten van mijn IC/BPS.'

- ☐ Mee eens
- ☐ Matig mee eens
- ☐ Onduidelijk
- ☐ Matig mee oneens
- ☐ Mee oneens

16. Geef aan in hoeverre u het eens bent met de volgende stelling:
'Door bacteriële blaasontstekingen moet ik veel vaker medische zorg zoeken voor mijn IC/BPS klachten.'

- ☐ Mee eens
- ☐ Matig mee eens
- ☐ Onduidelijk
- ☐ Matig mee oneens
- ☐ Mee oneens

Vragen over uzelf

Tot slot: Om de resultaten beter te kunnen analyseren stellen we u nog enkele vragen over uzelf. We zouden het op prijs stellen als u deze wilt beantwoorden. Bent u naaste van iemand met IC/BPS? Vul de laatste twee vragen dan in voor degene met IC/BPS.

148. Wat is uw geslacht?

- ☐ Man
- ☐ Vrouw
- ☐ Dat zeg ik liever niet
- ☐ Anders, namelijk ……………………………………………………………………

149. In welke leeftijdscategorie valt u?

- ☐ 0-18 jaar
- ☐ 19-40 jaar
- ☐ 41-60 jaar
- ☐ 61-80 jaar
- ☐ Ouder dan 80 jaar
